# School Environment and School Injuries

**DOI:** 10.3389/fpubh.2013.00076

**Published:** 2014-01-13

**Authors:** Simo Salminen, Marja Kurenniemi, Mirka Råback, Jaana Markkula, Anne Lounamaa

**Affiliations:** ^1^Finnish Institute of Occupational Health, Helsinki, Finland; ^2^National Research and Development Centre for Welfare and Health (STAKES), Helsinki, Finland; ^3^National Institute for Health and Welfare, Helsinki, Finland

**Keywords:** school injury, school yard, ice, snow, winter

## Abstract

**Background:** Although injuries at school are an important issue in public health, environmental factors in schools and school yards have seldom been the focus of school injury research. The goal of our investigation was to examine the effect of environmental factors on school injuries.

**Methods:** Nine comprehensive Finnish schools registered school injuries over a period of two school years. Injuries were classified as being associated with environmental factors, suspected environmental factors, and others. The consensus between two independent classifiers was 81%.

**Results:** A total of 722 injuries were classified. In 11.6% of these injuries, the physical environment factor was evident, and in 28.1% of the injuries, physical environment was suspected of being a contributory risk factor. Thus the physical environment of the school was a contributing factor in over a third (39.7%) of injuries occurring in the school, on the school yard or during the journey to, or from school. In this study, conducted in Finland, ice on the ground was mentioned most frequently as an environmental risk factor.

**Conclusion:** In Finland, the Nordic weather conditions are not taken into account in the school yard and playground plans as they ought to from the safety point of view. An initiative has been launched on a mandatory wintertime master plan for every school yard.

## Introduction

From the perspective of sociology, schools are one of the basic institutions in the society or perhaps as sub-systems of a society as Parsons (1) wrote. The fact is that schools are working in every society in the world. Thus the safety in schools is essential for the whole society.

In Finland, there are a total of 3026 primary schools, of which 3.1% were private schools and others are public schools. The average number of pupils was 175 (2). Finnish children between 10 and 14 years of age spent an average of 2 h and 22 min in schools every work day (3), whereas in Germany as much as 25–50% of their waking hours (4). Safety at schools is thus an important factor for public health.

Injuries at school are more common among boys than girls (5–9). However, girls are nearly twice as likely to be injured as boys at the school playground in Tucson, AZ, USA (10). In French schools, girls were injured during sports and physical training more often than boys (11), whereas in Swedish schools boys injured more often during sports and breaks (12).

Playgrounds exceeded all other sites of school injury for all age groups concluded a review of literature (6). However, another review showed that playground injuries were more frequent in elementary schools or kindergartens than in secondary schools (13). For instance, injury-risk situations occurred every 3 min among schoolchildren playing in a New England school playground. Boys were involved in these situations more often than girls, and were more likely to perform aggressive actions. Climbing and swinging equipments, in particular the slide, were identified as contributors to injury-risk situations (14). The injury rate at playgrounds was more than twice that of sports areas among, for example, Vancouver elementary school students (15). Fractures also occurred most often at playgrounds among elementary school children in Seattle (16), as did almost one fourth (23%) of school injuries in Alexandria, Egypt (17).

In Greek schools, playgrounds were dry during the occurrence of 74% of injuries, and the playground was covered by asphalt in 29% of cases (18). Head injuries were more frequent at the playground, whereas injuries to the lower and upper extremities were most frequent during sports in Swedish schools (19). There was a difference of up to 40 times in rates of equipment injury between schools in Tucson, AZ, USA (20). Playground equipment caused 38% of all playground injuries in Boulder Valley School District in Colorado (21) and the injury rate in Utah school playgrounds covered by asphalt was six times higher than that of those covered by sand (22). Hard surface on playground increased only boys’ activity (23). Playground injuries differed in their nature and body site affected from injuries sustained on the athletic field or in the gym (21).

Breaks and physical education lessons are school time which is spent on school grounds including playgrounds. A review showed that access to equipment, permanent play structure, and marked courts facilitated the physical activity of pupils during breaks (24). A total of 85% of injuries in Swedish schools occurred during breaks and physical education (25) which together comprised about one third of school time (26). The rate of injuries was also slightly higher in unorganized play or free time than during organized athletic programs in Hawaiian schools (27). In Poland, 37% of school injuries for pupils aged 7–15 years occurred during breaks, and 33% during physical education (28), while in Germany, 47% of school injuries were related to sports, 30% to playtime, and 17% to classroom lessons for all school-aged (29). Ball games and especially soccer has the greatest risk of school injury (29–31). Urban environment increased the risk of school injury both in Poland (28) and in China (32). Larger schools with equipment with full-size gymnasium had a higher school injury rate in Poland (28). On the contrary, in Japan the injury rate of pupils was highest in the small-sized schools (33).

Wintertime in Finland (December to February) is the most critical time period for school injuries. A previous study in the Turku region in Finland shows that most school injuries occurred in October and November and from January through March (34). This study revealed that the first ice storm before the real winter was also a critical time for injuries due to falls.

As the literature review showed together with activity, environmental factors are important potential causes of school injuries. However, the contribution of environmental factors to school injuries has not been studied yet except studies with playground equipments (20) and school environment checks in Sweden (35). The aim of this study was to examine the role of environmental factors in school injuries in Finland. This is done from the viewpoint of architecture, which means that the physical and built environment is the focal point. The built environment included playgrounds, its equipment, the floor, and playground cover, but not machines, balls, and moving equipment. It means also that school environment is looked at the point of planning.

## Materials and Methods

As a part of a larger project on school injuries in Finland (36), nine comprehensive schools from four Finnish cities registered injuries. The total number of pupils was 2900 and their age ranged from 7 to 15. The schools participated in this study on a voluntary basis and registered injuries over a period of two school years: 2002–2003 and 2003–2004. All injuries in which an injury pupil required the treatment of school nurse were included.

An “injury at school” was defined as either an intentional or unintentional injury that caused physical harm to the pupil. A teacher or school nurse filled in a school injury form (one A4 page) immediately after the injury, based on the injured pupil’s report. The form was completed for both injuries and violent acts, and the average time required to fill the form was 2 min. The form was same for all of the schools. It is partly based on the Swedish model (13).

The school injury form included a space for short description of the injury. During initial meetings with school staff and school nurses, researchers discussed how to write descriptions. The classification of the injuries as environmental factor, a suspected environmental factor, or a non-environmental factor as a cause is based on these written descriptions. The classification was carried out by two researchers independently (Marja Kurenniemi, Simo Salminen). The classification reliability was estimated by the consensus of the results of these two independent classifications. Their consensus based on the sample of 100 observations was 81.2%, which is above the acceptable level of 70% (37). Secondly, the reliability of classification was measured by Cohen’s coefficient of kappa (38). Its value was 0.50 (95% CI: 0.44–0.55) which was moderate agreement. In the case of inconsistency between classifiers the result of the main classifier (Marja Kurenniemi) was used.

An injury was classified as having been caused by an environmental factor where the effect of the environment was obvious and could be estimated to be the main reason for the injury occurrence: for example, pupil stepped on a door holder and fell. A suspected environmental factor concerned injuries in which the environmental factor was seen as a contributing factor but not an immediate cause: for example, the narrowness of a corridor contributing to the collision of pupils, causing injury. If the school environment has been different kind, the school children should behave in safe way in the situations where the injuries happened. Other injuries were classified as: no definite evidence that the environmental played a major role.

## Results

Over half of the injuries recorded in the registry occurred during breaks, and one out of four injuries during sports lessons. Boys were injured more often during breaks, whereas girls sustained injuries more often during sport lessons (36). Almost half (49%) of school injuries occurred outdoors. A total of 722 injury forms were classified. In 11.6% of classified injuries, a physical environment factor was evident, and was estimated to be the most significant factor contributing to the occurrence of the injury. In 28.1% of the injuries, the environment was considered an important and contributory risk factor. Thus the school environment was a contributing factor in 39.7% (287) of school injuries.

Table 1 shows the distribution of environmental factors in school injuries. One third of injuries were caused by slippery surfaces. School yard arrangements contributed to one fifth of injuries. Every 12th injury occurred in traffic environment. Other environmental factors had minimal influence on injuries. The following injuries are examples of a clear environmental factor: “A pupil fell down the stairs outside” (injury number 197), and “A pupil slipped and fell on his/her right hand” (injury number 431).

**Table 1:** **Environmental factors related to school injuries**.

Factor	Frequency	%	Typical injury
Door	7	2.5	Pinched fingers in the door
Chair	9	3.3	Coming down with a chair
Window	6	2.2	Sway and window get broken
Floor	4	1.4	Tumbled down on the roughness floor
Steps, handrails	6	2.2	Falling on steps with bad condition
Playgrounds	8	2.9	Get a piece of glass from playground
Equipments, slides	7	2.5	Jump over bicycle stand and injured his knee
Traffic arrangements	24	8.7	Crash with bicycle to a car
School yard arrangements	59	21.3	Fall by sand on the asphalt
Space arrangements	12	4.3	Fall on the bench of the corridor
Slipping	12	4.3	Slipping and falling during the play
Slippery	90	32.5	Slipping on icy school ground
Others	33	11.9	Football hit to student’s forehead

Total	277	100.0	

**Figure 1:**
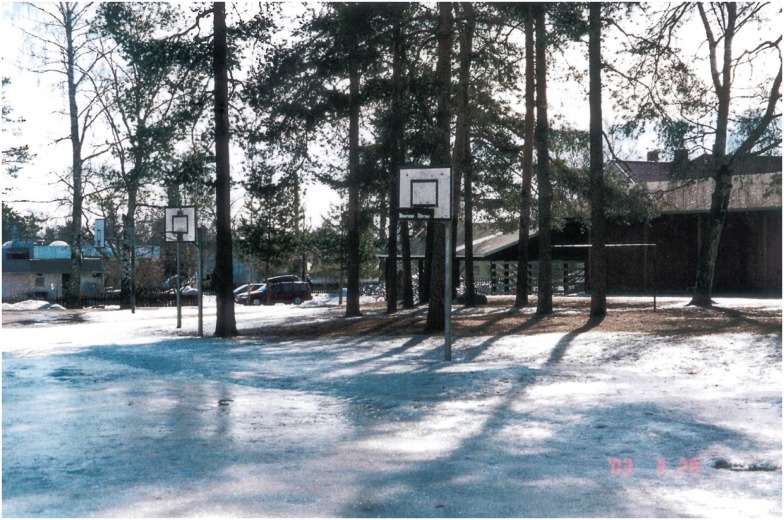
**The Finnish school yard during the wintertime**.

An example of a contributing factor is: “A pupil rode his/her bicycle to the end of the asphalt and fell” (injury number 720). Another injury where the environment was a contributing factor is: “A pupil’s left shoulder rubbed against the railing in the swimming pool causing a bruise” (injury number 643). Ice was the most frequently mentioned environmental factor (in 5.8% of injuries) associated with school injuries in the school yard. For example: “A pupil fell on the icy school ground, breaking one front tooth and splitting a lip” (injury number 42). An example for icy school playground is shown in Figure 1.

## Discussion

One of the main conclusions of this study is that in Finland, the architecture of school yards and playgrounds are designed for the summertime, and are not suitable during wintertime, when there is snow and ice on the ground. This explains why there are so many falls in Finnish schools.

Finnish children have long summer vacations, from June to mid-August, and attend school during the winter months. The snow and icy season varies depending on the location of the school: in southern Finland, the season is on average from November to March, whereas in the north it lasts from October to April.

Maintaining school yards during the winter months, recognizing ice as a significant risk factor, following the weather forecast for icy days, and passing on this information to maintenance personnel, could reduce school injuries in countries such as Finland. The results of this study led to a mandatory wintertime master plans for school yards in Finland including first elimination of ice and snow from the school yard and then sanding the yard. The high compensation of school injuries [even $ 50,000 Ref. (39)] increased the meaning of the injury prevention.

Another method to prevent injuries at school yards is the safety round method (40). The head master and a group of teachers and pupils walk around the school area and especially school yard observed the risk places of injuries. The important point is the participation of pupils, because they see risks from different perspectives than adults. This safety check should be done annually (41). Importance of this perspective is emphasized by the Lithuanian study with schoolchildren showing that feeling unsafe at school increased the risk of injury (42). Pupils’ perception of low justice increased the risk of being dissatisfied with school and absence due to truancy (43).

Rigorous, effective injury prevention efforts at school should address several factors: the environment, individual behavior, social norms, legislation, and policy. Improvements to the physical environment of the school through regular safety assessments, good quality maintenance, and repairing hazards immediately after they are identified, can contribute to school safety. To tackle these challenges, attention should be paid to both organizational and everyday routine practices in schools. In this way, we can guarantee children’s rights to a safe environment – a safe school environment.

Environmental modification and increased supervision can reduce school injuries (44). However, increased teacher supervision not necessarily help to prevent injuries, as 88% of injuries at Missouri schools, for instance, occurred while pupils were allegedly supervised by adults. Supervisor of school children is especially difficult during lunch break (45). High supervision increased both boys’ and girls’ physical activity at school yards in San Diego (46). On the other hand, a playground injury prevention plan (47) based on the S.A.F.E. model (48) could modify the school yard safer. Schools need also after-school program to prevent injuries in their playgrounds after the school hours (49).

The most important limitation of this study is that the schools participated in this study on a voluntary base. We cannot be sure that they reported all the injuries in their school. For example, Scottish schools under-reported injuries, even those requiring hospital treatment (50). In Canada, schools routinely reported only one out of five injuries and one out of two serious injuries (51). In Wales, a third of primary schools did not report their injuries to the authorities (52). We assume that the schools in this study also under-reported their injuries, but we cannot estimate the extent of this suspected under-reporting.

The other limitation of this study is that all participating schools situated in the cities. However, majority of 4300 Finnish primary schools worked at the country side, but they have at minimum one teacher and 5–10 pupils. Thus the unknown number of school injuries is a problem only in big schools and in the cities.

## Conflict of Interest Statement

The authors declare that the research was conducted in the absence of any commercial or financial relationships that could be construed as a potential conflict of interest.
